# A novel luciferase-based assay for the detection of Chimeric Antigen Receptors

**DOI:** 10.1038/s41598-018-38258-z

**Published:** 2019-02-13

**Authors:** Ramakrishnan Gopalakrishnan, Hittu Matta, Sunju Choi, Venkatesh Natarajan, Ruben Prins, Songjie Gong, Arta Zenunovic, Nell Narasappa, Fatima Patel, Rekha Prakash, Vishan Chaudhary, Varun Sikri, Saurabh Deepak Chitnis, Andrei Kochegarov, Dan Wang, Magdalena Falat, Michael Kahn, Pooja Smruthi Keerthipati, Naman Sharma, Jyotirmayee Lenka, Tomas Meza Stieben, Jason Braun, Ankita Batra, Katelyn Purvis, Kenta Ito, Jae Han Lee, Alberto Jeronimo, Hannalei Mae Zamora, Allen Membreno, Queenie Qiu, Supriya Peshin, Lalith Namburu, Preet M. Chaudhary

**Affiliations:** Jane Anne Nohl Division of Hematology and Center for the Study of Blood Diseases, University of Southern California, Keck School of Medicine, Los Angeles, California, United States of America

## Abstract

Chimeric Antigen Receptor-T (CAR-T) cell immunotherapy has produced dramatic responses in hematologic malignancies. One of the challenges in the field is the lack of a simple assay for the detection of CARs on the surface of immune effector cells. In this study, we describe a novel luciferase-based assay, termed Topanga Assay, for the detection of CAR expression. The assay utilizes a recombinant fusion protein, called Topanga reagent, generated by joining the extra-cellular domain of a CAR-target in frame with one of the marine luciferases or their engineered derivatives. The assay involves incubation of CAR expressing cells with the Topanga reagent, a few washes and measurement of luminescence. The assay can detect CARs comprising either immunoglobulin- or non-immunoglobulin-based antigen binding domains. We further demonstrate that addition of epitope tags to the Topanga reagent not only allows its convenient one step purification but also extends its use for detection of CAR cells using flow cytometry. However, crude supernatant containing the secreted Topanga reagent can be directly used in both luminescence and flow-cytometry based assays without prior protein purification. Our results demonstrate that the Topanga assay is a highly sensitive, specific, convenient, economical and versatile assay for the detection of CARs.

## Introduction

Chimeric Antigen Receptor (CAR) therapy is a revolutionary approach for the treatment of human malignancies. Generally, a CAR is engineered by fusing in-frame the single chain variable fragment (scFv) of a monoclonal antibody to a module containing a hinge domain, a transmembrane domain and one or more signaling domains. Boosted by the recent approvals of CD19-CAR for B-cell acute lymphoblastic leukemia and refractory diffuse large B-cell lymphoma the field is moving forward at a rapid pace. As such, the number of clinical trials using CAR therapy for various human malignancies is growing rapidly.

A major challenge in the CAR field, however, is the lack of a fast, economical, sensitive, and robust assay for the detection of CARs on the surface of immune effector cells. Expression of CARs on effector cells is generally detected by flow cytometry using fluorochrome-tagged antibodies or ligands that bind to the extra-cellular domain of the CAR^1–3^. Most of the detection antibodies, however, are polyclonal and suffer from lot-to-lot variations that may lead to inconsistent results. CD19-specific CARs have been detected following staining with an Alexa Flour 488-conjugated CD19-Fc fusion protein consisting of human CD19 extracellular domain and Fc region of human IgG1^3^. This protocol, however, requires the extra steps and costs associated with fusion protein purification and its conjugation with Alexa Fluor 488^3^. CAR-expressing T cells have also been detected using biotinylated Protein L^2^. Staining using biotinylated Protein L necessitates additional protocol steps of secondary staining with labeled streptavidin, which may lead to potential loss of cells^3^. Although some CARs can be detected using anti-idiotype antibodies (e.g. CD19), such antibodies are available for only FMC63 antibody based CARs and are not available for other CARs^1^. All the above methods need flow cytometry for read out. Further, many of them utilize a secondary labeling step for detection, which is time consuming and labor intensive.

Luciferases have been extensively used in biomedical research due to their ability to provide highly sensitive quantitation with low background^4,5^. Firefly luciferase (Fluc) is one of the most popular luciferase in research, and has a MW of 61 kDa. The large size of Fluc, however, has hampered its use in fusion protein studies. Recently, several marine luciferases have been discovered from deep sea organisms, that are smaller in size (approximately 19 kDa) and are much brighter than Fluc^4,6^.

In this study, we describe the development of a novel luciferase-based assay for the detection of CAR expression on the surface of immune cells. The assay utilizes a recombinant fusion protein, called Topanga reagent, which is generated by joining the extra-cellular domain of a CAR target in frame with one of the marine luciferases. As they use marine luciferases, the assay and the reagent were named after the Topanga Beach in Los Angeles, California. The word Topanga is Native American in origin and means “where the mountain meets the sea”.

## Results

### Development of a luciferase-based method for the specific detection of CAR

**Figure 1:**
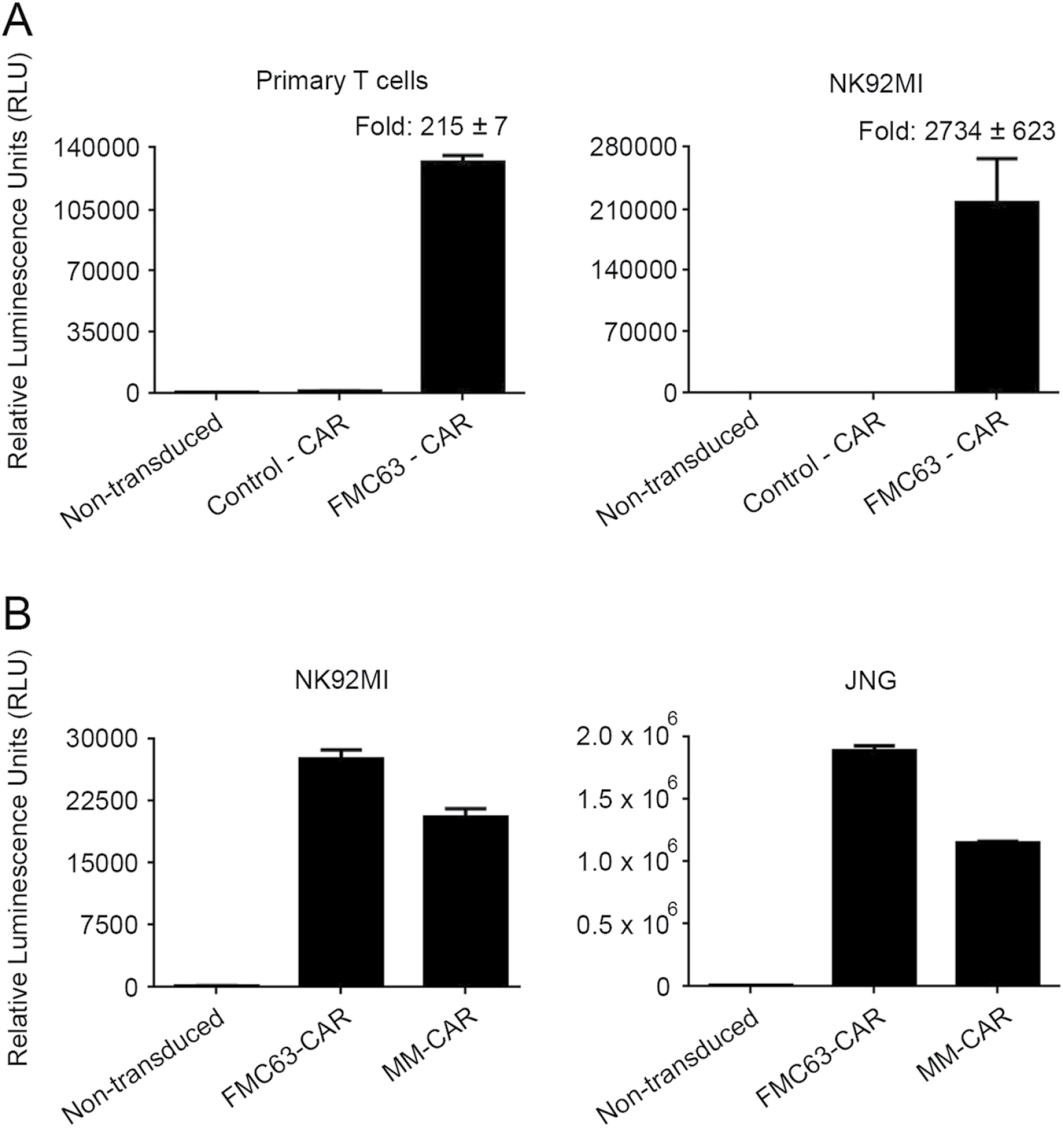
Development of Topanga assay for the detection of Chimeric Antigen Receptors (CAR). (**A**) Approximately, 2 × 10^5^ human primary T and NK92MI cells expressing the indicated CARs were incubated for an hour with 100 µl of supernatant containing CD19-ECD-Nluc fusion protein. After incubation and washing steps, luminescence was measured by addition of Coelenterazine-containing assay buffer. (**B**) Topanga assay performed as described in (**A**) using NK92MI cells or JNG cells expressing the indicated CD19 CARs. The values shown are mean ± SE of a representative of at least two experiments performed in duplicate.

Recently discovered/engineered marine luciferases such as Gluc, Nluc, Tluc16, and Mluc7 are smaller in size (approximately 19 kDa) than the more commonly used firefly luciferase (61 kDa)^4^. Further, these marine luciferases are 1000-fold brighter and more stable than firefly luciferase^4,7,8^. To develop Topanga assay for the detection of CD19 CARs, we made a fusion construct by joining in frame the extracellular domain (ECD) of human CD19 containing a signal peptide with Nluc via an intervening short Gly-Gly-Ser-Gly flexible linker. The fusion construct was transiently transfected into 293FT cells. The supernatant containing the secreted Topanga reagent (i.e., CD19-ECD-Nluc fusion protein) was collected approximately 48 hours after transfection and used to bind primary T and NK92MI cells stably expressing a CD19-CAR. This CAR, designated FMC63-CAR, contained the antigen binding domain derived from a scFv (single chain variable fragment) based on murine FMC63 antibody. T cells and NK92MI cells stably expressing a CD33-CAR were used as negative controls. As shown in Fig. 1A, the CD19-CAR-expressing T and NK92MI cells showed marked increase in luminescence when bound by the CD19-ECD-Nluc fusion protein as compared to the control CAR (i.e. CD33-CAR)-expressing cells or parental (non-transduced) cells. These results demonstrated the specificity of the CD19-ECD-Nluc Topanga reagent for detecting the expression of CD19-CAR expressing cells and confirmed that a luciferase-based assay can be developed for the specific detection of CARs.

While all the FDA-approved CARs are based on FMC63, CARs based on antigen binding domains derived from scFvs other than FMC63 are in clinical development. We next examined whether the Topanga assay can be utilized to detect the expression of CD19-CARs containing antigen binding domains derived from scFvs other than FMC63. For this purpose, NK92MI and JNG cells stably expressing CD19-CARs containing scFv sequences derived from a mouse monoclonal antibody against human CD19 (Clone MM) were bound to CD19-ECD-Nluc fusion protein, followed by luminescence detection. As shown in Fig. 1B, there was a robust increase in the luminescence values in wells that contained NK92MI and JNG cells expressing CD19 targeting CARs in comparison to non-transduced cells. These results suggest that a single marine luciferase fusion construct containing the full extracellular domain of CD19 can be used to detect cells expressing different CD19-CARs.

### Use of Tluc16, Mluc7 and Paluc1 in Topanga assay

**Figure 2:**
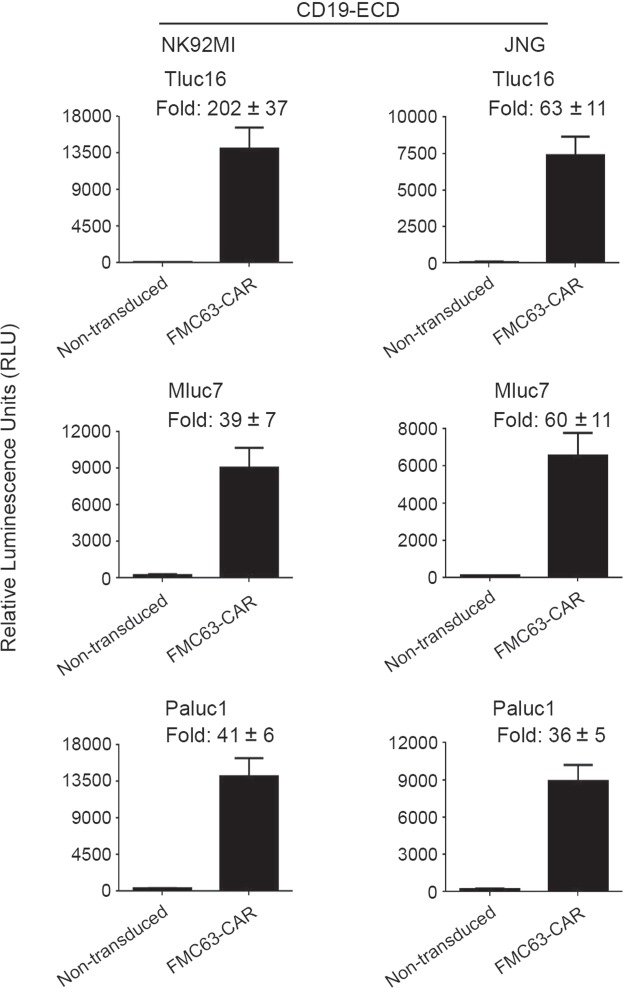
NK92MI and JNG cells stably expressing a FMC63-CAR and non-transduced cells were incubated for 1 hour with 100 µl of supernatants containing the CD19-ECD-Tluc16, Mluc7 or Paluc1 fusion proteins. After incubation, cells were washed 5 times and assayed for luminescence.

In addition to Nluc, a number of marine luciferases, such as Tluc16, Mluc7, and Paluc have been described^6,9^. To determine whether these marine luciferases can be used in place of Nluc to generate Topanga reagents, we made fusion constructs that expressed the CD19-ECD in fusion with Tluc16, Mluc7 or Paluc1, respectively. The fusion proteins were produced by transient transfection of the corresponding constructs in 293FT cells and tested for binding to CD19-CAR-expressing NK92MI and JNG cells. The CD19-CAR-expressing NK92MI and JNG cells showed a dramatic increase in the luminescence activity when bound by all three CD19-ECD-fusion proteins as compared to the non-transduced NK92MI and JNG cells (Fig. 2). Thus, potentially any marine luciferase can be used as a fusion partner for the generation of Topanga reagent for use in the Topanga assay.

### Topanga assay can be used to detect CARs other than CD19 CARs

**Figure 3:**
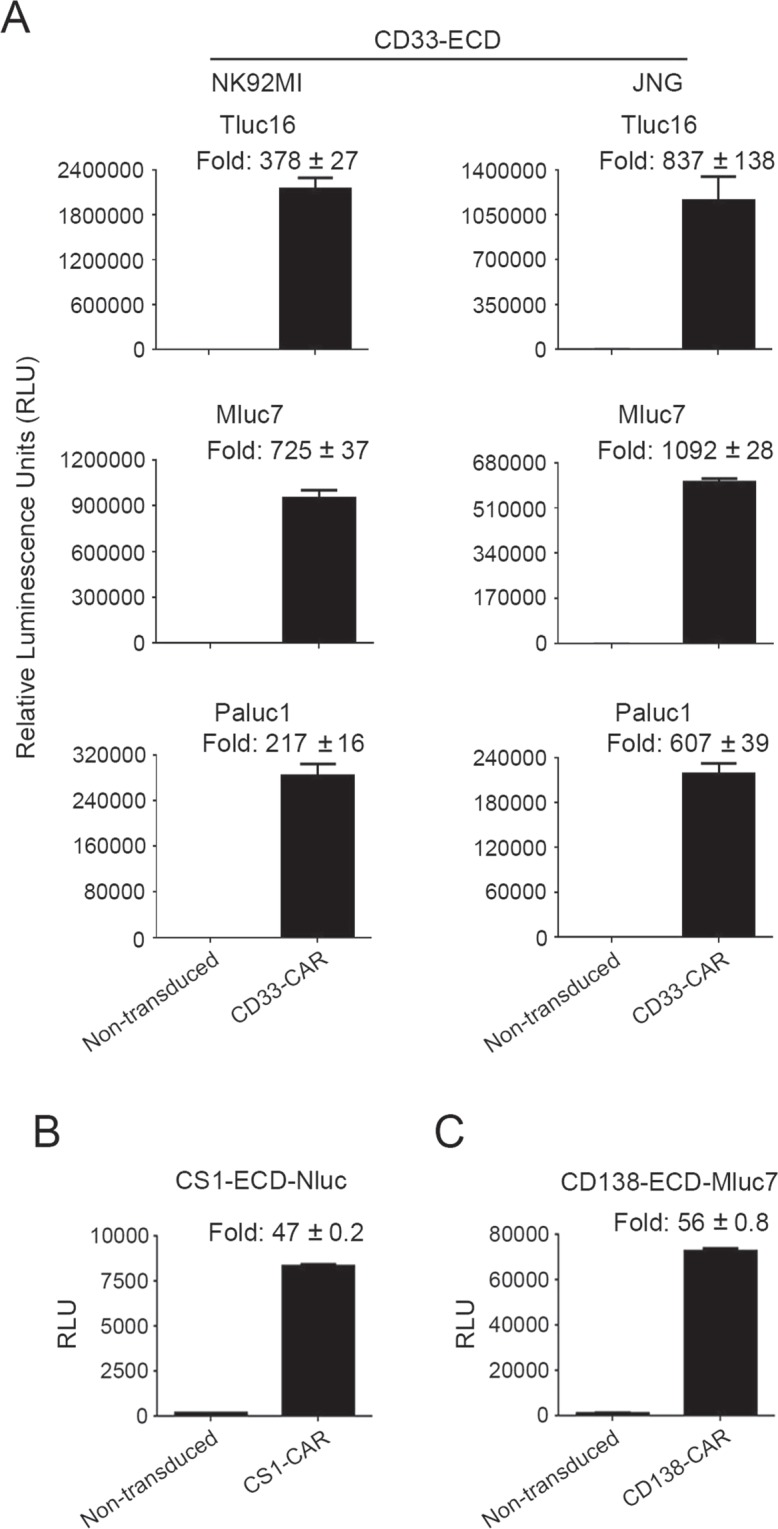
(**A**) NK92MI and JNG cells stably expressing a CD33-CAR and non-transduced cells were incubated for an hour with 100 µl of supernatants containing the CD33-ECD-Tluc16, Mluc7 or Paluc1 fusion proteins. After incubation, the cells were washed and assayed for luminescence. (**B**) Topanga assay was performed as described above with non-transduced NK92MI cells and those stably expressing a CS1-CAR using the CS1-ECD-Nluc fusion protein. (**C**) Topanga assay was performed with the non-transduced NK92MI cells and those stably expressing a CD138-CAR using the CD138-ECD-MLuc7 fusion protein. The values shown are mean ± SE of a representative of at least two experiments performed in duplicate.

We next tested the ability of the Topanga assay to detect the expression of CARs targeting antigens other than CD19. CARs targeting CD33 are being developed for the treatment of myeloid malignancies^10,11^. We made constructs containing the extracellular domain of human CD33 in fusion with Tluc16, Mluc7 or Paluc1 and tested the ability of the fusion proteins to bind to NK92MI and JNG cells stably expressing a CD33 CAR. As shown in Fig. 3A, CD33-CAR-expressing NK92MI and JNG cells showed a dramatic increase in luminescence activity when bound by any of the three CD33-ECD-fusion proteins as compared to the non-transduced cells.

The surface antigen CS1 (also known as CD319 or SLAMF7) is a marker of normal plasma cells and malignant plasma cells in multiple myeloma^12^. Similarly, CD138 (also known as Syndecan 1) is highly expressed in normal and malignant plasma cells^13^. CARs targeting CS1 and CD138 are in development for the treatment of plasma cell disorders^14,15^. We generated CS1-ECD-Nluc and CD138-ECD-Mluc7 fusion proteins and tested their binding to NK92MI cells stably expressing CARs targeting CS1 and CD138, respectively. Binding of CS1-ECD-Nluc, and CD138-ECD-Mluc7 to their corresponding CAR expressing cells resulted in 47 and 56-fold increase in luminescence values, respectively, as compared to non-transduced cells (Fig. 3B,C). Taken collectively, the above results demonstrate that the Topanga assay has broad applicability for the detection of CARs.

### Topanga assay can be used to detect non-scFv based CARs

**Figure 4:**
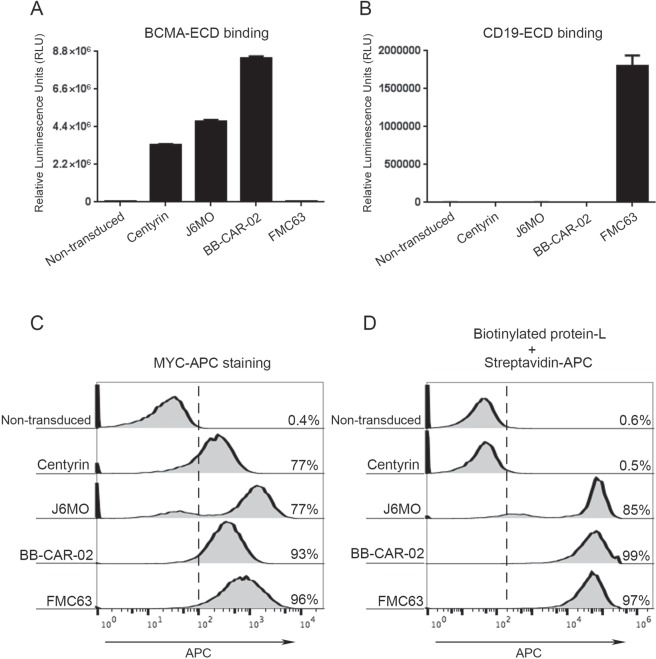
(**A**) Non-transduced JNG cells and JNG cells stably expressing the indicated BCMA-CARs (Centyrin, J6MO, and BB-CAR-02) and a CD19-CAR (FMC63) were incubated with 100 µl of supernatant containing the BCMA-ECD-Nluc fusion protein for 45 minutes on ice. After incubation, cells were washed 5 times and assayed for luminescence. (**B**) The assay was performed as described in (**A**) except that CD19-ECD-Nluc fusion protein was used. (**C**) JNG cells stably expressing the indicated CARs were incubated with an APC-conjugated MYC antibody for 45 minutes on ice. After incubation, cells were washed twice and analyzed by flow cytometry. (**D**) JNG cells stably expressing the indicated CARs were stained with Protein L reagent as described in methods section followed by flow cytometry analysis. The values shown are mean ± SE of a representative of at least two experiments performed in duplicate.

Recently, non-scFv based CARs have been developed using antibody mimetics^16,17^. For example, CARs based on Centyrins, a novel class of non-immunoglobulin antigen binding scaffold protein based on a consensus fibronectin domain, are being developed^18^. One of the traditional methods to detect CARs relies on the ability of Protein L to bind to the V_L_ region of scFvs. However, Protein L is not expected to bind to non-scFv based CARs. To test whether the Topanga assay can detect non-scFv based CARs, a BCMA targeted non-scFv based CAR derived from a Centyrin was developed. Two CARs based on scFv derived from BCMA antibodies (Clones J6MO and BB-CAR-02) were also constructed to serve as positive controls. Finally, an FMC63 scFv based CD19 CAR was used as a negative control. All the CAR constructs were stably transduced into JNG cells. As shown in Fig. 4A, JNG cells stably expressing the Centyrin-based and the scFv-based BCMA-CARs showed significant increases in the luminescence when bound by BCMA-ECD-Nluc fusion protein as compared to the non-transduced cells or the JNG cells expressing the FMC63-based CD19 CAR. In contrast, CD19-ECD-NLuc fusion protein bound only to the CD19-CAR (FMC63) expressing JNG cells and showed no significant binding to non-transduced or BCMA-CAR expressing cells, thereby again confirming the specificity of the Topanga assay (Fig. 4B). All the above CAR constructs expressed a MYC-tag which was located immediately downstream of their antigen binding domain. As expected, JNG cells stably expressing BCMA- and CD19-targeted CAR were readily detected by flow cytometry using a MYC antibody (Fig. 4C). However, Protein L could not detect the expression of the Centyrin-based non-scFv BCMA CAR (Fig. 4D). Taken collectively, the above results demonstrate that the Topanga assay can detect any CAR irrespective of the nature of its antigen binding domain and thus has an advantage over the assay based on Protein L.

### Topanga assay is a highly sensitive assay

**Figure 5:**
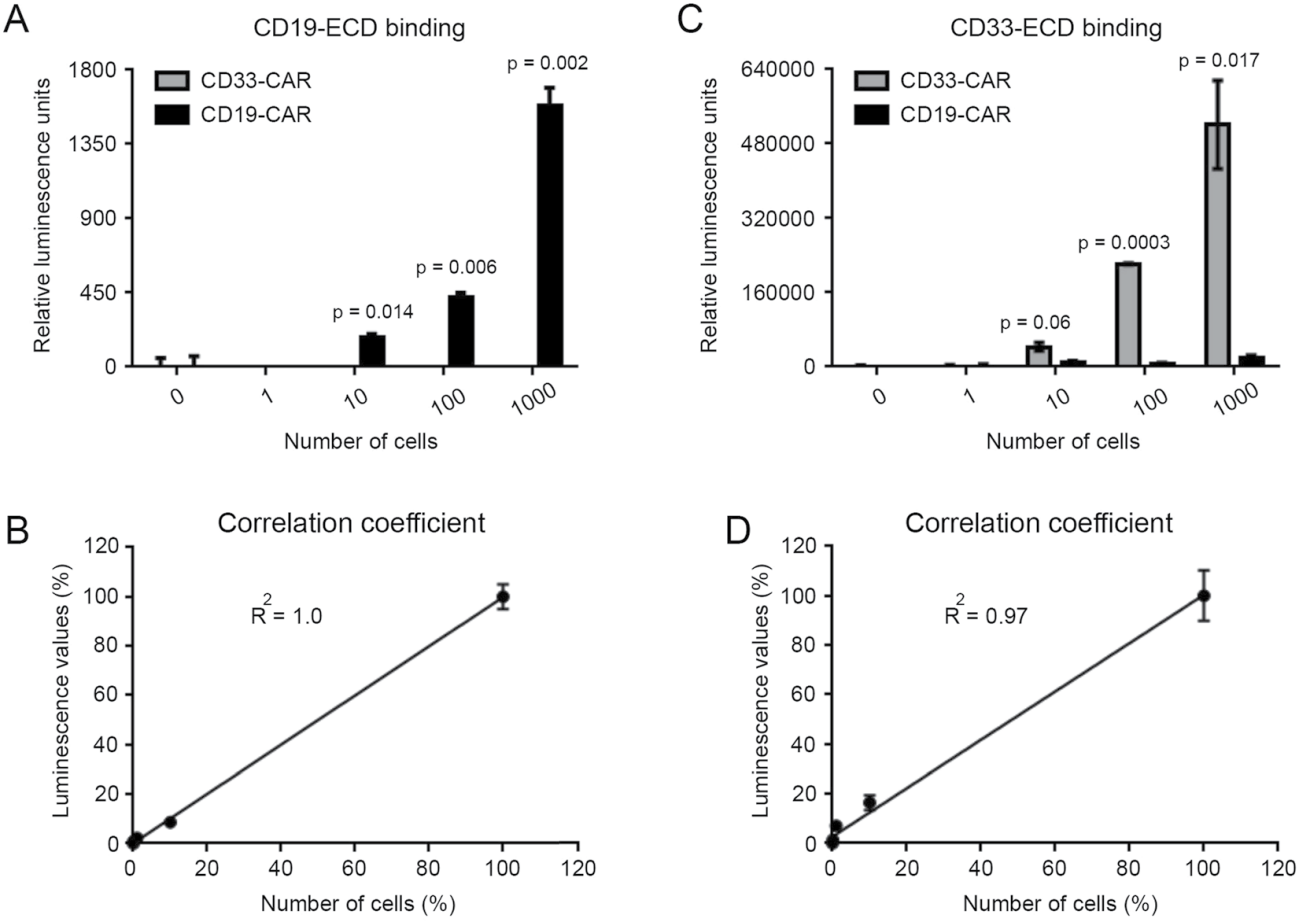
(**A**) Human primary T cells were transduced with a lentiviral vector encoding a CD19-CAR (FMC63) that also expresses a puromycin resistance gene (Pac). Transduced cells were selected with puromycin (400 ng/mL) for 2 weeks to obtain a pure population of CAR-T cells. Indicated number of puromycin-selected CAR-T cells were mixed with 1 × 10^6^ PBMCs, incubated with 100 µl of supernatant containing the CD19-ECD-Nluc fusion protein and subjected to the Topanga assay. (**B**) Linear increase in luminescence over a wide range of cell numbers. Both the number of CAR-T cells plated and luminescence values detected were converted into percentage by dividing the individual values with the maximum number of cells plated (10000) or the luminescence values from the wells with maximum cell number. (**C**,**D**) The experiment was performed as described for A and B, except cells stably expressing an CD33-CAR and CD33-ECD-Tluc16 fusion protein were used. R^2^ = Correlation coefficient. The values shown are mean ± SE of a representative of at least two experiments performed in duplicate.

To determine the sensitivity of the Topanga assay, different numbers of primary T cells stably expressing a CD19-specific CAR or a CD33-specific CAR were mixed with 1 million PBMCs followed by binding assay with the CD19-ECD-Nluc fusion protein. A linear increase in luminescence value was seen with an increase in cells expressing CD19-specific CAR (R^2^ = 1) (Fig. 5A,B). Importantly, a significant increase in luminescence was observed even in wells that contained only ten CD19 CAR-positive cells when bound with the CD19-ECD-Nluc fusion protein (Fig. 5A). In contrast, the luminescence values in wells containing increasing numbers of CD33-specific CAR stayed close to the baseline, demonstrating the specificity of the assay (Fig. 5A). Essentially similar results were obtained when primary T cells stably expressing a CD19-specific CAR or a CD33-specific CAR were mixed with 1 million PBMCs followed by a binding assay with the CD33-ECD-Tluc16 fusion protein (Fig. 5C,D). Collectively, the above results highlight the sensitivity, specificity and broad linearity of the Topanga assay for the detection of CAR-expressing cells.

### Topanga assay can be completed in 30 minutes without any loss in specificity

**Figure 6:**
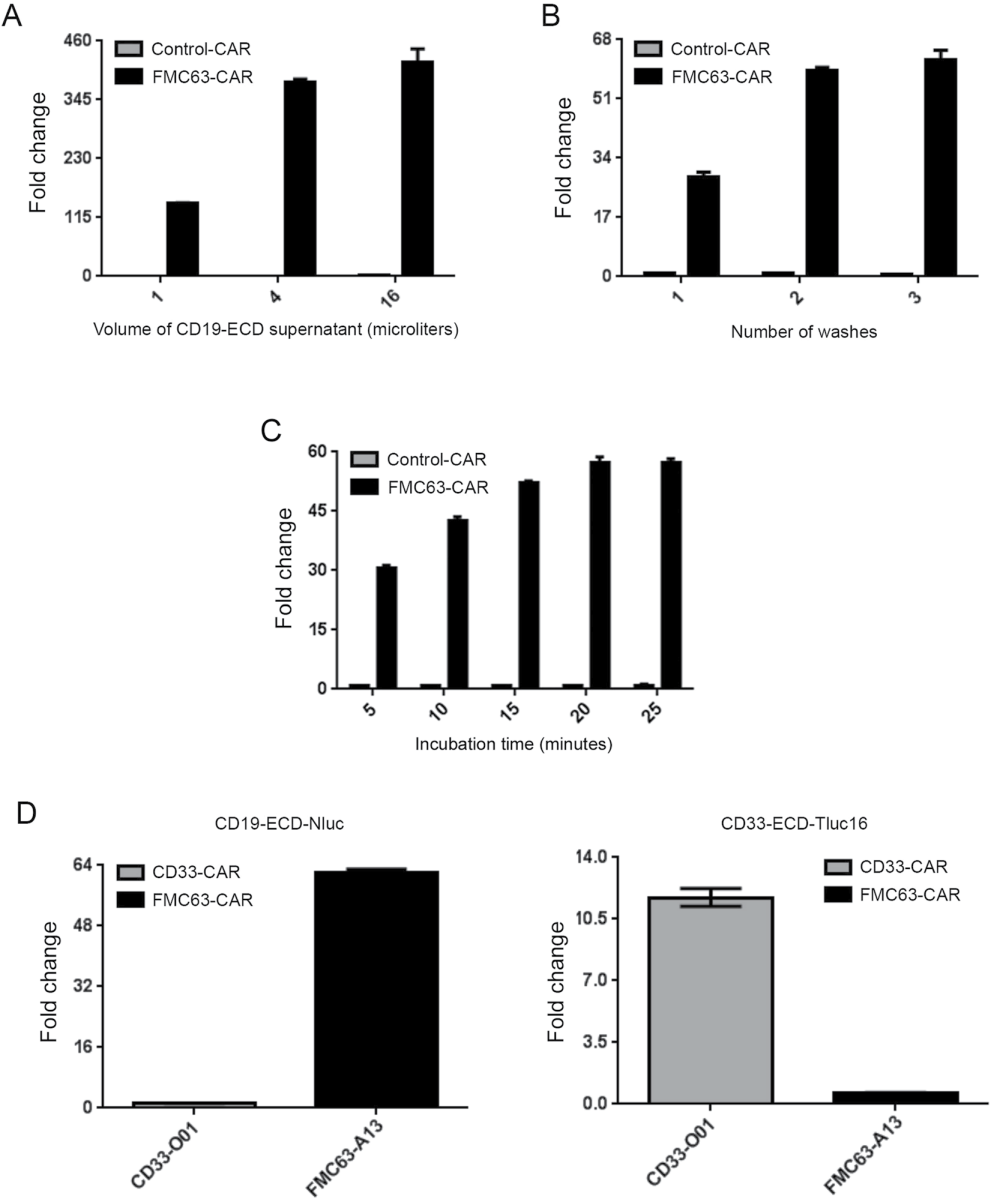
Topanga assay can be completed in 30 minutes. (**A**) Non-transduced NK92MI cells and NK92MI cells stably expressing the indicated FMC63-CAR and CD33-CAR (Control) were incubated with the indicated volumes of supernatant containing the CD19-ECD-Nluc fusion protein for 45 minutes on ice. After incubation, cells were washed 5 times and assayed for luminescence. The data was presented as fold change in comparison to luminescence values in the wells of non-transduced cells. (**B**) Topanga assay was performed as described for (**A**) except that only 1 µl of supernatant was used. Post incubation, the cells were washed 1, 2 and 3 times and assayed for luminescence. (**C**) The experiment is similar to as described in (**B**) except that the cells were incubated for indicated times and post-incubation the cells were washed twice and assayed for luminescence. (**D**) Human primary T cells transduced with FMC63-CAR, CD33-CAR and non-transduced cells were incubated with 1 µl of the indicated supernatants for 20 minutes on ice. Post incubation, the cells were washed twice and assayed for luminescence. A representative of two independent experiments is shown.

The Topanga assay described in the preceding sections included an incubation step of 45 minutes and 5 washes and took approximately 70 minutes to complete. To develop an optimized protocol for the Topanga assay, we began by examining the minimum amount of the CD19-ECD-Nluc supernatant that is sufficient for the performance of the assay. For this purpose, NK92MI cells stably expressing an FMC63-CAR were incubated with different volumes of supernatant containing the CD19-ECD-Nluc fusion protein. NK92MI cells stably expressing a control-CAR and non-transduced NK92MI cells were included as negative controls. NK92MI cells expressing the FMC63-CAR showed an approximately 142-fold increase in luminescence over the control CAR-expressing cells when bound by only 1 µl of CD19-ECD-Nluc supernatant and there was a progressive increase in fold change in luminescence with increase in the volume of the supernatant (Fig. 6A). Next, we tested the optimum number of washes required for the Topanga assay when using 1 µl of the CD19-ECD-Nluc supernatant. As shown in Fig. 6B, following a single wash, CD19-CAR-expressing NK92MI cells showed an approximately 27-fold increase in luminescence over control cells. Whereas, there was a greater fold increase in luminescence after 2^nd^ and 3^rd^ washes (approximately 59 and 62 respectively), this was primarily due to a reduction in the non-specific luminescence (Fig. 6B). From the above experiments, we decided to use 1 µl of CD19-ECD-Nluc supernatant and 2 washes for the Topanga assay. Finally, we tested the incubation time needed for the Topanga assay. For this purpose, 1 µl of CD19-ECD-Nluc supernatant was bound to NK92MI cells stably expressing a CD19-CAR or control cells for 5, 10, 15, 20, and 25 minutes, followed by two washes and measurement of luminescence. While an incubation period of only 5 minutes showed a 31-fold increase in luminescence, there was a linear increase in luminescence fold change with increase in incubation times up to 20 minutes (Fig. 6C). Collectively, the above results demonstrate that the Topanga assay can be completed in as little as 30 minutes, including an incubation period of 20 minutes and two washes. Essentially similar results were obtained when the experiment was repeated with primary T-cells stably expressing a CD19-CAR or a CD33-CAR (Fig. 6D).

### Topanga assay can be coupled with flow cytometry for detection of CARs

We added epitope tags to the fusion proteins to facilitate their purification. For example, a 4xFlag-2xStreptagII-8xHis cassette carrying 4 copies of a FLAG epitope tag, 2 copies of a StreptagII and a polyhistidine tag was added to the carboxy-terminus of CD19-ECD-TLuc16 via an intervening Glycine-Serine (GGSG) linker. The addition of the epitope tags had no adverse effect on the expression, secretion or antigen binding ability of the fusion proteins (data not shown). We purified the CD19-ECD-Tluc16-4xFlag-2xStreptag-8xHis fusion protein by affinity chromatography using a Strep-Tactin column. The purified protein was active, as determined by a specific binding assay on T cells and NK91M cells expressing the FMC63-CAR (Supplementary Fig. S1). Furthermore, using the purified CD19-ECD-Tluc16-4xFlag-2xStreptag-8xHis fusion protein, we determined that Topanga assay can be successfully performed with as little as 31 ng/mL of the protein (Supplementary Fig. S2).

**Figure 7:**
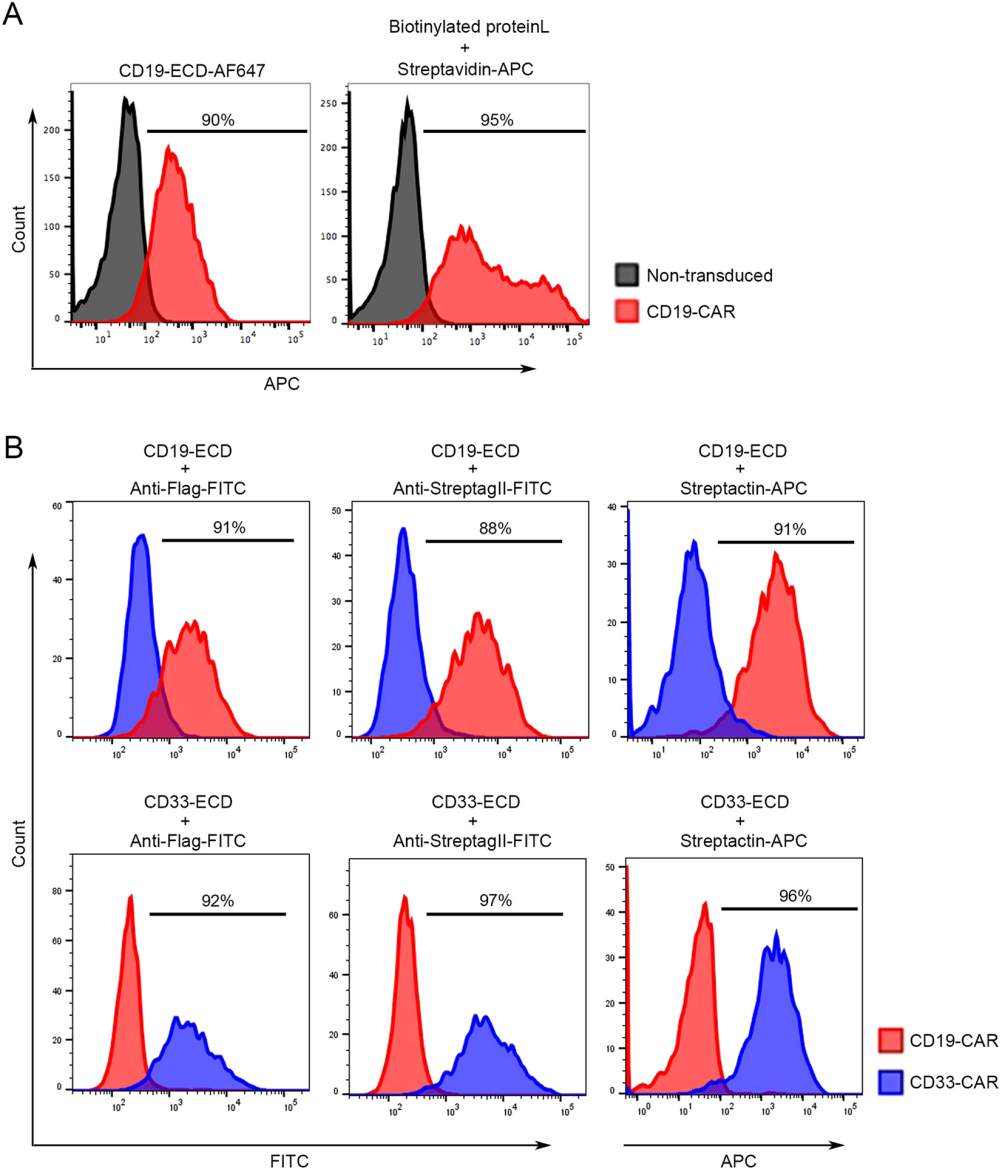
(**A**) Human primary T cells transduced with a CD19-CAR and parental cells were stained with purified CD19-ECD fusion protein labelled with Alexa Fluor- 647 (AF647). Alternatively, the cells were labeled with Biotinylated Protein L followed by APC-conjugated Streptavidin. After washes, cells were analyzed by flow cytometry. (**B**) Human primary T cells expressing a CD19-CAR or a CD33-CAR were incubated with 100 µl of supernatants containing the epitope-tagged CD19- or CD33-ECD fusion proteins for 45 minutes on ice. After incubation, cells were washed 3 times and incubated with the indicated anti-tag antibodies for 45 minutes. After 2 washes, cells were analyzed by flow cytometry. A representative of two independent experiments is shown.

A major limitation of luminescence based Topanga assay is that it does not provide information about the percentage of CAR-expressing cells. To determine if the epitope-tagged Topanga reagents can be also used to determine percentage of CAR-expressing cells, the purified CD19-ECD-Tluc16-4xFlag-2xStreptag-8xHis fusion protein was labelled with Alexa Fluor 647 (AF-647) and tested for its ability to detect CD19-specific CAR cells using a one-step flow cytometry-based method. For this purpose, primary T cells stably expressing a CD19-specific CAR or non-transduced T cells were incubated with CD19-ECD-AF647 for 45 minutes, followed by washing and detection by flow cytometry. As shown in Fig. 7A, CD19-CAR-T cells showed a significant increase in fluorescence as compared to non-transduced cells upon incubation with CD19-ECD-AF647. Most importantly, the percentage of CD19-specific CAR cells detected by flow cytometry using the single-step method using CD19-ECD-AF647 Topanga reagent and a multi-step method using Biotinylated Protein L reagent were comparable. Thus, the Topanga reagents developed for the luminescence-based Topanga assay can be also used for the detection and isolation of CARs using flow cytometry.

We next examined if the crude epitope-tagged Topanga reagents can be used for detection of CAR-expressing cells using flow cytometry without going through the time-consuming and costly steps of protein purification and labeling. For this purpose, T cells stably expressing a CD19-CAR and a CD33-CAR were labelled with CD19-ECD-Tluc16-4xFlag-2xStreptag-8xHis and CD33-ECD-Tluc16-4xFlag-2xStreptag-8xHis fusion proteins. After washes, cells were labelled with FITC-conjugated antibodies against FLAG or StreptagII or with Strep-Tactin-APC, which binds with high affinity to the StreptagII. As shown in Fig. 7B, CD19-CAR-T cells could be specifically detected by primary staining with the CD19-ECD-Tluc16-4xFlag-2xStreptag-8xHis fusion protein followed by secondary staining with FITC-conjugated Flag or StreptagII antibodies or with APC-conjugated Strep-Tactin. Essentially similar results were obtained when the CD33-ECD-Tluc16-4xFlag-2xStreptag-8xHis fusion protein was used to detect CD33-CAR-T cells (Fig. 7B, lower panel).

## Discussion

Assays for detection of CARs are needed for the research and development of novel CAR constructs, in the CAR-manufacturing, and for monitoring the persistence of CARs after administration to the patients. Although a number of assays are in current use for the detection of CARs, all of them suffer from major limitations. For example, quantitative PCR or RT-PCR are frequently used to monitor persistence of CAR in the patients^19,20^. However, these assays are time-consuming, expensive and prone to contamination errors. As the PCR-based assays do not measure the CAR at the protein level, they do not provide any information about the functional expression of the CAR. A number of flow cytometry based assays have been described for the detection of CARs on the cell surface^1–3^. However, these assays are generally time consuming, require purified protein reagents and a flow cytometer. The Topanga assay described here is the first of a kind assay to utilize marine luciferases for the detection of CAR expression. The assay has several advantages over the existing assays, including high sensitivity, specificity, convenience, speed and low-cost.

The high sensitivity of the Topanga assay is partly due to the utilization of the recently discovered novel marine luciferases, which are usually 1000-fold brighter than the most commonly used firefly luciferase^4,7,8^. We were able to detect as few as 10 CAR-expressing cells in the background of 1 million peripheral blood mononuclear cells using crude supernatant of fusion constructs-transfected 293FT cells. While we have not used the Topanga assay for detecting CAR-T cells in clinical samples, we believe that it would have a similar sensitivity for the detection of CAR-T cells from the blood of patients receiving this therapy. It is conceivable that the sensitivity of the assay can be improved further using purified and concentrated fusion proteins and upon further optimization of the staining protocol.

We further demonstrate that the Topanga assay is highly specific with extremely low non-specific binding. This is in contrast to other assays for CAR-detection that have been reported in the literature, such as staining with Protein L or CD19-Fc fusion protein^2,3^. The high specificity and low-background of the Topanga assay may be partly due to the fact that it involves a single binding step as compared to other assays. In addition, the Topanga reagent lacks an Fc module, which could potentially contribute to non-specific binding to cells containing the Fc receptors. In contrast to assays utilizing CD19-Fc or anti-idiotype antibodies, the Topanga assay does not involve any secondary antibody labeling, which further reduces non-specific binding and contributes to its high specificity. Although CARs can be detected with relatively little non-specific binding by the use of good quality anti-idiotype antibodies, such antibodies are available for only a few CAR constructs. For example, to the best of our knowledge, anti-idiotype antibodies have been only described for the detection of FMC63-based CD19 CARs^1^ and are not available against most, if not all, other CARs. In contrast to Protein-L or anti-idiotype antibody based flow-cytometry assays, the Topanga assay is an assay of functional expression of CAR that also provides information about the ability of the CAR to bind to its target. Finally, another advantage of the Topanga assay is its ability to detect a CAR irrespective of the nature of its antigen binding domain.

Other major benefits of the Topanga assay are its relatively low cost. All currently available assays for detection of CARs at the protein level require the use of expensive instruments, such as a flow cytometer^1–3^. Data acquisition and analysis using a flow cytometer, however, is not only time consuming but also requires special training. While it may be true that investigators working in the CAR field in major research institutions in the developed countries have access to a flow cytometer, the CAR field is rapidly expanding to areas beyond major research institutions in the developed countries where the access to a flow cytometer may be limited. Furthermore, even at major research institutions, operating a flow cytometer and data analysis either requires special training or the use of a core facility, where access is often limited to the working hours and is charged an hourly rate. In contrast, Topanga assay can be performed using a luminometer, a relatively inexpensive equipment, which is available in most laboratories and does not require special training. The cost is further reduced by the facts that, if needed, it can be performed using crude supernatants without the time-consuming and expensive steps of protein purification and labeling as the label (i.e. luciferase) is incorporated in the fusion protein itself. Finally, the relatively low cost of the luciferase substrate (i.e., coelenterazine) contributes to the reduced cost of the assay.

The speed of the Topanga assay is another major advantage. We have determined that the whole assay can be completed in 30–40 minutes which compares favorably to the 2–3 hours needed for a typical flow cytometry experiment. We have successfully optimized all steps of the Topanga assay, including sample binding, washes and measurement of luminescence, and validation in a 96 well format (data not shown) to further reduce the assay time, especially when multiple samples have to be processed. Due to its extreme sensitivity and lack of operator dependence for interpretation of results, the Topanga assay is also ideally suited for miniaturization and automation. Further, high stability of the marine luciferases at room temperature and under cell culture conditions makes the Topanga Assay amenable to High Throughput Screening (HTS) applications^4^.

A disadvantage of the Topanga assay, as described herein, as compared to the Protein-L based detection assay is the need to prepare the Topanga reagents specific for different CAR targets, e.g., CD19, BCMA and CD138. However, with the advances in synthetic biology, this is not a major impediment and we were able to generate expression constructs encoding Topanga reagents specific for a number of CAR constructs in a relatively short time. Once the expression constructs are prepared, the recombinant Topanga reagents can be expressed relatively easily by transient transfection into suitable cells, e.g., 293FT cells. Furthermore, supernatant containing the secreted protein can be used in the Topanga assay directly without the need of laborious and time-consuming steps of protein purification and labeling as are needed for other assays. The secreted Topanga reagents can be also frozen at −80 °C for later use without significant loss of activity.

In comparison to the flow cytometry based assays, the Topanga luciferase assay does not directly provide information regarding the percentage of CAR-expressing cells. This information, however, may not be critical in all research and/or clinical applications. Nevertheless, we demonstrate that various epitope tags can be added to the luciferase fusion proteins and such epitope-tagged Topanga reagents can be used for the detection of CAR-expressing cells using flow cytometry. Thus, with a modest investment of time and money in the generation of the Topanga reagent, a research laboratory can have access to both luminometer- and flow cytometer-based assays for CAR detection. Topanga assay can be used as a stand-alone assay for many research and clinical application. Furthermore, it can be used in combination with other assay, for example, to confirm the results obtained with a flow-cytometry based assay, to rule out non-specific binding, and to provide information regarding the binding affinity of the different CAR constructs. Therefore, Topanga reagents described in this report are versatile reagents that can be used for detection of CAR-expressing cells using both luciferase-based and flow cytometry-based assays.

We demonstrate that the marine luciferase containing fusion proteins can be purified without any loss of activity. We also conjugated the purified Tluc16 fusion protein to Alexa Fluor 647. However, the Alexa Fluor 647-conjugated Tluc16 protein showed diminished luciferase activity, although it did retain its binding to CAR, thereby allowing only a flow cytometry-based detection. It is possible that conjugating fluorophores using other available conjugation methods may retain the enzymatic activity of the luciferases. Alternatively, this problem can be solved by selecting a marine luciferase that does not lose its enzymatic activity upon conjugation to fluorophores.

The Topanga assay has several potential applications in the field of cellular immunotherapy. The assay can be used to compare the expression of different CAR constructs and to select the CAR constructs with the desired expression level for further development and clinical use. The assay can be potentially used during manufacturing of cell therapy products as a quality-control and product-release assay. Finally, studies are also being planned to test the utility of the assay for monitoring the expansion and persistence of CAR-T cells after their administration in patients.

## Materials and Methods

### Cell lines and reagents

NK92MI cells were obtained from ATCC and maintained as per the instructions provided. JNG (**J**urkat cell line engineered with a **N**FAT-dependent E**G**FP reporter gene) was kindly provided by Dr. Arthur Weiss (UCSF) and maintained in RPMI-1640 medium supplemented with 10% FBS^21^. 293FT cells were obtained from Invitrogen (ThermoFisher Scientific) and cultured as recommended. Primary T cells were isolated and cultured as described earlier^4^. Polybrene and coelenterazine were obtained from Sigma and Nano light technology, respectively. Strep-Tactin-APC (IBA-lifescience: Cat# 6-5010-001); Anti-Flag-FITC (Sigma: F4049); Anti-Strep-tagII-FITC (Genscript: A01736); Biotinylated Protein L (Genscript: M0097); Streptavidin-APC conjugate (Molecular probes: SA1005); APC-conjugated anti-MYC antibody (R&D systems: IC3696A); and Polyethylenimine (Polysciences Inc: 23966) were obtained from the indicated sources.

### Construction of lentiviral vectors encoding marine luciferase fusion proteins

The nucleotide sequences encoding the extra-cellular domain (ECD) of human CD19, CD33, CS1, CD138 and BCMA were fused in frame to human codon-optimized cDNAs encoding signal peptide-deleted variants of marine luciferases (e.g., Nluc, Tluc16, Mluc7, and Paluc1) via an intervening short Glycine-Serine flexible linker. All the constructs carried an N-terminal signal peptide, either native to the receptor or derived from human CD8, to allow secretion of the fusion protein. The resulting gene fragments were cloned in the pLenti-EF1α lentiviral expression vector^4^. In some cases, the fusion proteins also carried C-terminal epitope tags, such as FLAG, FLAGx3, Strep-tagII, 8xHis, AcV5, HA or a combination of several tags. Some constructs also co-expressed a puromycin resistance gene (PAC, Puromycin Acetyltransferase), which was separated from the expression cassette encoding the luciferases fusion proteins by a 2A ribosomal skip sequence.

### Construction of lentiviral based chimeric antigen receptors (CARs), preparation of lentivirus supernatants, and generation of stable cell lines expressing CARs

The lentiviral vector encoding FMC63-BBz CAR has been described previously^4^. This CAR carried a MYC epitope between the scFv region and the human CD8 transmembrane domain^4^. CARs targeting CD33, CS1, CD138 and BCMA were constructed by replacing the FMC63 scFv targeting CD19 with publically available scFvs targeting different antigens. In the CD33 CAR construct, the 41BB costimulatory domain was replaced by a CD28 costimulatory domain. Lentiviral infection and generation of primary T cells, NK92MI and JNG expressing the different CARs were performed as described previously^4^.

### Production of Topanga reagents

The Topanga fusion proteins were produced in 293FT cells by transient transfection of different expression constructs using calcium phosphate or polyethylenimine methods^22^. In general, 10 µg of each fusion protein expression plasmid and 0.25 µg of EGFP (Enhanced Green Fluorescent Protein) encoding plasmid were used for each 100-mm plate. Medium was changed 16 hours post-transfection in case calcium phosphate was used for transfection. Approximately 48 hours after transfection, supernatants were collected, filtered through a 0.45 µm filter, and stored at −80 °C in aliquots until further use.

### Binding assay and luminescence detection

Approximately 2 × 10^5^ CAR-expressing cells were used per reaction. The cells were spun down at 1300 rpm for 5 minutes, re-suspended in 100 µl of supernatants containing the fusion proteins and incubated on ice for 45 minutes. Post-incubation, cells were washed 5 times with 1 ml of ice cold wash buffer (0.5% FBS in PBS). After the final wash, the pellet was re-suspended in 30 µl wash buffer and added to a white 384-well Lumitrac plate. Coelenterazine (CTZ) was used as a substrate to measure luminescence using a CTZ-assay buffer containing 20 µM CTZ in PBS^4^. Unless indicated otherwise, the assay buffer with the substrate was added to cells at 1:1 or 1:4 ratio (v/v) for all luciferase assays. Luminescence was read in an endpoint mode using a BioTek synergy H4 hybrid microplate reader for 10 seconds.

### Purification of hCD19-ECD-Tluc16-fusion protein and labelling with Alexa Fluor® 647

The expression construct encoding hCD19-ECD-Tluc16 fusion protein was transiently transfected in to 293FT and supernatant was collected as described earlier. Approximately, 500 ml of supernatant was used to purify the fusion protein as described in the manual for “Gravity flow Strep-Tactin® XT Superflow® column − 10 ml” (iba-lifesciences.com: 2-4014-001). The purified protein was labelled with Alexa Fluor^TM^ 647 microscale protein labeling kit (ThermoFisher Scientific: A30009) as described previously^23,24^. The labelled protein was referred as CD19-ECD-AF647.

### Flow cytometry

Cells were bound with 100 µl of supernatants containing Topanga reagents with multiple epitope tags as described under binding assay. After washes, the cells were incubated with fluorophore-labelled anti-tag antibodies (Anti-Flag, Anti-StreptagII) or Strep-Tactin for 45 minutes on ice followed by 3 washes with wash buffer. For CD19-ECD-AF647 staining, cells were incubated with 0.1 µg of CD19-ECD-AF647 for 45 minutes on ice followed by 3 washes with wash buffer. Finally, the cells were re-suspended in 200 µl wash buffer and analyzed by flow cytometer (BD FACSVerse^TM^). The FACS data was analyzed using FlowJo. MYC staining was performed using an APC-conjugated anti-MYC antibody. Protein L staining was performed as described earlier^2^.

### Statistical analysis

Two-tailed unpaired Student *t* test was used to test for differences between 2 groups using GraphPad Prism 5 software. Differences with a *P* ≤ 0.05 were considered statistically significant. All experiments were repeated a minimum of two times.

## Supplementary information

Topanga- Supplementary information

## Data Availability

The data and reagents will be available up on request to senior author P.M.C.
