# Supplementary material for: A novel luciferase-based assay for the detection of Chimeric Antigen Receptors
Source: Sci Rep. 2019 Feb 13;9:1957. doi: 10.1038/s41598-018-38258-z (PMC6374361; doi:10.1038/s41598-018-38258-z)
Supplement: Supplementary file 1 — Topanga- Supplementary information [file 41598_2018_38258_MOESM1_ESM.docx]

**A novel luciferase-based assay for the detection of Chimeric Antigen Receptors**

Ramakrishnan Gopalakrishnan**^*^**, Hittu Matta**^*^**, Sunju Choi**^*^**, Venkatesh Natarajan**^*^**, Ruben Prins, Songjie Gong, Arta Zenunovic, Nell Narasappa, Fatima Patel, Rekha Prakash, Vishan Chaudhary, Varun Sikri, Saurabh Deepak Chitnis, Andrei Kochegarov, Dan Wang, Magdalena Falat, Michael Kahn, Pooja Smruthi Keerthipati, Naman Sharma, Jyotirmayee Lenka, Tomas Meza Stieben, Jason Braun, Ankita Batra, Katelyn Purvis, Kenta Ito, Jae Han Lee, Alberto Jeronimo, Hannalei Mae Zamora, Allen Membreno, Queenie Qiu, Supriya Peshin, Lalith Namburu and Preet M Chaudhary^#^

*These authors contributed equally to this work.

Jane Anne Nohl Division of Hematology and Center for the Study of Blood Diseases, University of Southern California, Keck School of Medicine, Los Angeles, California, United States of America.

^#^**Corresponding author:** Preet M. Chaudhary, M.D., Ph.D.

**Email:** preet.chaudhary@med.usc.edu; **Phone:** 323-865-3916; Fax: 323-865-0060.

**Supplementary Information**

**
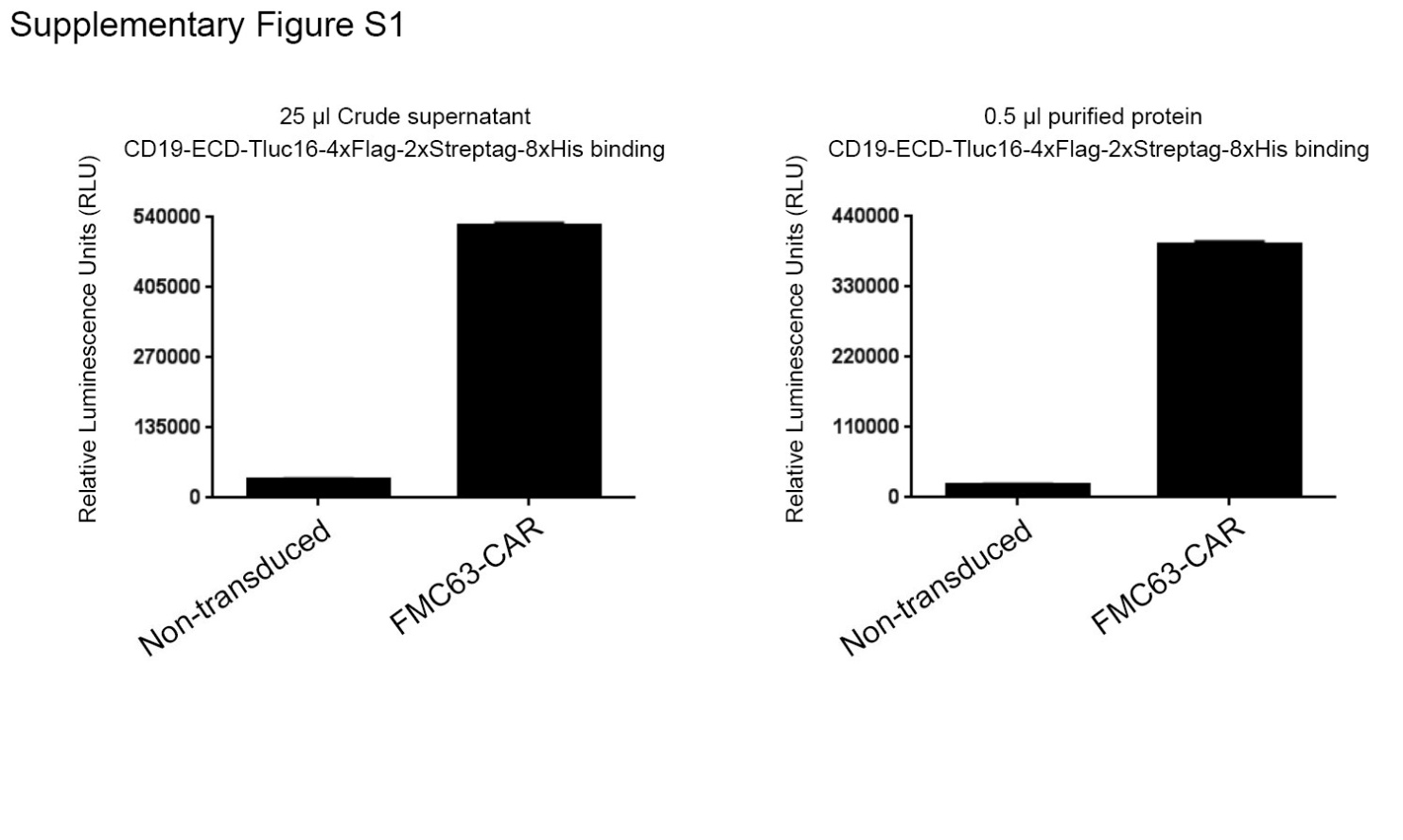
**

**Supplementary Fig. S1.** Purified CD19-ECD-Tluc16 fusion protein retains its activity. Approximately 2 x 10^5^ human primary T cells stably expressing a CD19 specific FMC63-CAR were incubated with 25 µl of crude supernatant containing the CD19-ECD-Tluc16-4xFLAG-2xStreptagII-8xHis fusion protein or 0.5 µl of purified CD19-ECD-Tluc16-4xFLAG-2xStreptagII-8xHis fusion protein for 45 minutes on ice. After incubation, cells were washed 5 times and assayed for luminescence by addition of coelenterazine-containing assay buffer (25 µl) directly to each well in a 384-well Lumitrac plate.

**
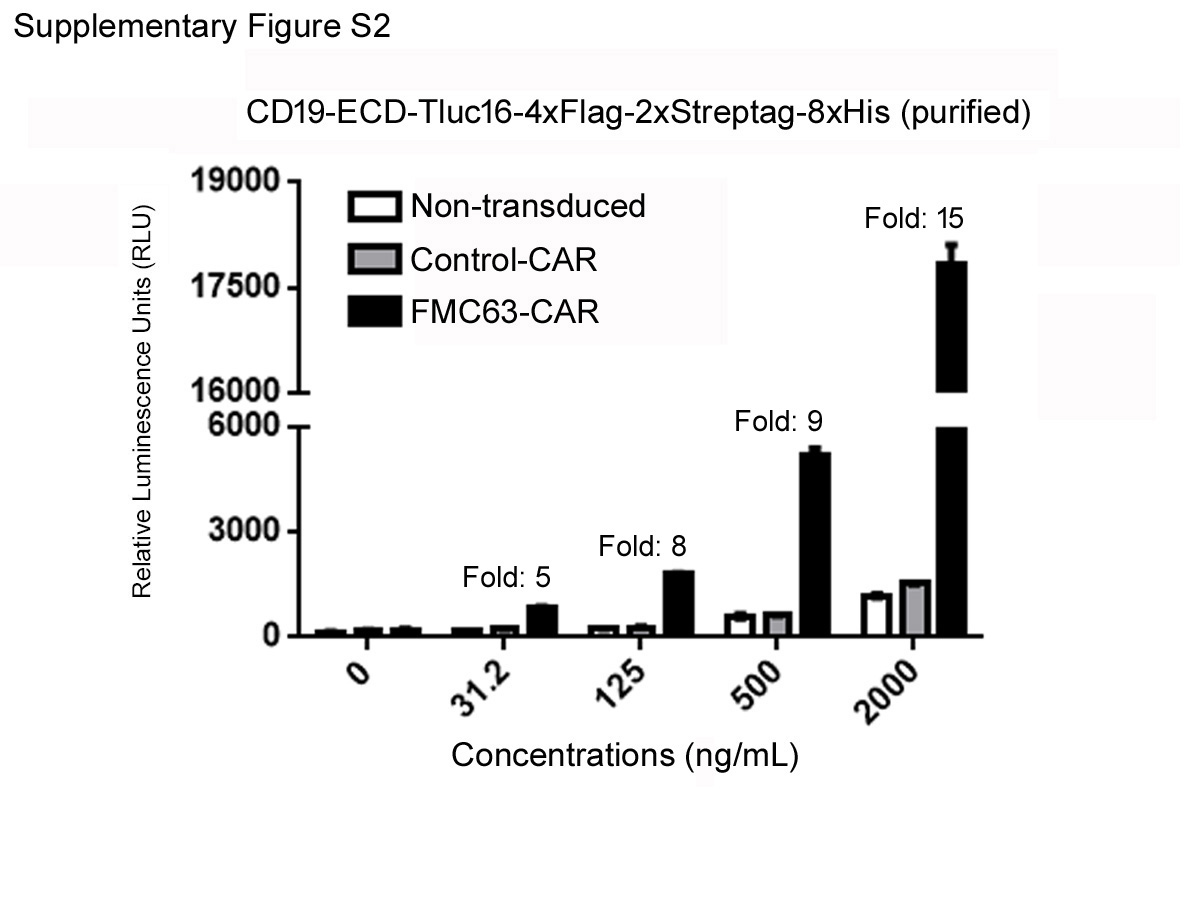
**

**Supplementary Fig. S2.** Performance of Topanga assay using purified CD19-ECD-Tluc16 fusion protein. Approximately 2 x 10^5^ non-transduced NK92MI cells and NK92MI cells stably expressing the indicated FMC63-CAR and CD33-CAR (negative control) were incubated with the indicated concentrations of purified CD19-ECD-Tluc16-4xFLAG-2xStreptagII-8xHis fusion protein for 45 minutes on ice. After incubation, cells were washed 5 times and assayed for luminescence by addition of coelenterazine-containing assay buffer (25 µl) directly to each well in a 384-well Lumitrac plate.
